# Identification of Heterotrimeric G Protein γ3 Subunit in Rice Plasma Membrane

**DOI:** 10.3390/ijms19113591

**Published:** 2018-11-14

**Authors:** Aki Nishiyama, Sakura Matsuta, Genki Chaya, Takafumi Itoh, Kotaro Miura, Yukimoto Iwasaki

**Affiliations:** Department of Bioscience and Biotechnology, Fukui Prefectural University, 4-1-1 Kenjojima, Matsuoka, Eiheiji-Town, Fukui 910-1195, Japan; s1873016@g.fpu.ac.jp (A.N.); s1873018@g.fpu.ac.jp (S.M.); s1873012@g.fpu.ac.jp (G.C.); ito-t@fpu.ac.jp (T.I.); miura-k@fpu.ac.jp (K.M.)

**Keywords:** GS3, γ subunit, heterotrimeric G protein, mass spectrometric analysis, RGG3, rice, western blotting

## Abstract

Heterotrimeric G proteins are important molecules for regulating plant architecture and transmitting external signals to intracellular target proteins in higher plants and mammals. The rice genome contains one canonical α subunit gene (*RGA1*), four extra-large GTP-binding protein genes (XLGs), one canonical β subunit gene *(RGB1*), and five γ subunit genes (tentatively named *RGG1*, *RGG2*, *RGG3/GS3/Mi/OsGGC1*, *RGG4/DEP1/DN1/OsGGC3*, and *RGG5/OsGGC2*). *RGG1* encodes the canonical γ subunit; *RGG2* encodes the plant-specific type of γ subunit with additional amino acid residues at the N-terminus; and the remaining three γ subunit genes encode the atypical γ subunits with cysteine abundance at the C-terminus. We aimed to identify the *RGG3/GS3/Mi/OsGGC1* gene product, Gγ3, in rice tissues using the anti-Gγ3 domain antibody. We also analyzed the truncated protein, Gγ3∆Cys, in the *RGG3/GS3/Mi/OsGGC1* mutant, *Mi*, using the anti-Gγ3 domain antibody. Based on nano-liquid chromatography-tandem mass spectrometry (LC-MS/MS) analysis, the immunoprecipitated Gγ3 candidates were confirmed to be Gγ3. Similar to α (Gα) and β subunits (Gβ), Gγ3 was enriched in the plasma membrane fraction, and accumulated in the flower tissues. As *RGG3/GS3/Mi/OsGGC1* mutants show the characteristic phenotype in flowers and consequently in seeds, the tissues that accumulated Gγ3 corresponded to the abnormal tissues observed in *RGG3/GS3/Mi/OsGGC1* mutants.

## 1. Introduction

Heterotrimeric G proteins are well known to consist of three subunits, α, β, and γ, in mammals and yeast [1,2,3,4]. Receptors regulating the heterotrimeric G proteins, such as G protein-coupled receptors (GPCRs), interact with external signals and activate the heterotrimeric G proteins via the intrinsic GDP/GTP exchange factor (GEF) of GPCRs. When GTP binds to the α subunit (Gα-GTP), heterotrimeric G proteins dissociate into the α subunit (Gα-GTP) and βγ dimer. The α subunit and βγ dimer can regulate respective effector molecules. Thus, heterotrimeric G proteins are signal mediators from receptors to effector molecules. In higher plants, heterotrimeric G proteins are important molecules for regulating plant architecture and transmitting external signals to intracellular target proteins [5,6,7]. The biochemical characteristics of the plant heterotrimeric G protein [5] and the signaling mechanism and effector molecules regulating the plant heterotrimeric G protein [6] have been previously reviewed. The plant morphology of heterotrimeric G protein mutants has also been previously summarized [7]. There are three extra-large GTP-binding protein genes (*AtXLG1*~*AtXLG3*) [8,9], one canonical α subunit gene (*GPA1*) [10], one canonical β subunit gene (*AGB1*) [11], and three γ subunit genes (*AGG1*~*AGG3*) [12,13,14], in *Arabidopsis*; and four extra-large GTP-binding protein genes (prediction by in silico) [15], one canonical α subunit gene (*RGA1*) [16], one canonical β subunit gene (*RGB1*) [17], and five γ subunit genes, which we tentatively named *RGG1* [18], *RGG2* [18], *RGG3/GS3/Mi/OsGGC1* [19], *RGG4/DEP1/DN1/OsGGC3* [20], and *RGG5/OsGGC2* [21], in this paper.

With regard to the γ subunit genes in *Arabidopsis*, there are *AGG1* and *AGG2* encoding the canonical γ subunits, and *AGG3* encoding the atypical γ subunit with cysteine abundance at the C-terminus. In rice, *RGG1* encodes the canonical γ subunit, *RGG2* encodes the plant-specific type of γ subunit, and the remaining three γ subunit genes, *RGG3/GS3/Mi/OsGGC1*, *RGG4/DEP1/DN1/OsGGC3*, and *RGG5/OsGGC2* encode the atypical γ subunits homologous to *AGG3*. *RGG3* corresponds to *GRAIN SIZE 3 (GS3)* [19] and *RGG4* corresponds to *DENSE AND ERECT PANICLES 1* (*DEP1/DN1*) [20]. The genome sequence of *RGG5* was predicted by Botella [21]. The diversity and agronomical importance of plant γ subunits have been previously reviewed [21,22].

Mutants of *XLG1*, *XLG2*, and *XLG3* [23]; *GPA1* [24]; *AGB1* [25,26]; and *AGG1*, *AGG2* [27], and *AGG3* [14] were isolated as heterotrimeric G protein mutants in *Arabidopsis*. Mutants of *RGA1* [28,29], *GS3* [30], and *DEP1* [20] were isolated as similar G protein mutants in rice. By morphological analysis of *gpa1* [24], *agb1* [26], *d1* [31], and RGB1 knock-down lines [32], it was shown that plant heterotrimeric G proteins modulate cell proliferation.

It has been shown that plant heterotrimeric G proteins are associated with transduction in response to multiple external signals, namely auxin [24,26], abscisic acid [33,34,35,36,37], gibberellin [38,39,40,41], brassinosteroid [24,39,40], sugar [42,43], blue light [44,45], and ozone [46]. It was also shown that the heterotrimeric G proteins of plants are concerned with defense signaling [47,48,49,50].

Based on the characteristics of heterotrimeric G proteins in higher plants, the α subunit is suggested to be contained in a huge complex localized in the plasma membrane fraction of rice [18] and *Arabidopsis* [51]. In rice, some βγ dimer candidates seem to be present in two different forms: one is a component of a huge complex, and the other is a sole βγ dimer dissociated from a huge complex in the plasma membrane of rice seedlings [18]. Using yeast two-hybrid screening, it was shown that 68 highly interconnected proteins form the core G-protein interactome in *Arabidopsis* [52], in which the regulators of G protein signaling protein (AtRGS1) [53], THYLAKOID FORMATION 1 (THF1) [43], cupin domain protein (AtPrin1) [35] etc. in addition to α, β, γ1, γ2 subunits, were contained. The huge complexes prepared solubilized plasma membrane fraction in rice [18] and *Arabidopsis* [51] may represent a part of the G-protein interactome in *Arabidopsis* [52]*.*

In mammals and yeast, β subunits interact with γ subunits to form the βγ dimer [1,2,3,4]. The βγ dimer has not been purified from the tissues of higher plants so far, but many studies suggest its presence based on the experiments, including an in vitro pull-down assay [12,13], yeast two-hybrid (Y2H) assay [13], split-ubiquitin system [14], and fluorescence response energy transfer (FRET) assay [51,54] in *Arabidopsis*. Moreover, in rice, the β subunit was shown to interact with the γ1 and γ2 subunits with a Y2H assay [18]. Recently, the interaction of rice β subunit with atypical γ subunits and the localization of these subunits in the plasma membrane were demonstrated with a bi-molecular fluorescence complementation (BiFC) assay [55,56]. These results indicated that both the canonical and atypical γ subunits can interact with the β subunit, and that βγ dimers are localized in the plasma membrane fraction, in *Arabidopsis* and rice.

*GS3* is identified as a major QTL for grain weight and grain length, and as an important gene for agriculture [19,30,56,57,58]. According to the identification of *AGG3* in *Arabidopsis*, *GS3* was classified as the atypical γ subunit member, and tentatively named *RGG3*. In order to understand the mechanism of seed formation in rice, studies on the GS3 protein are important.

To understand the function of *RGG3* in the regulation of seed size, identifying the native Gγ3 protein is important. When the native Gγ3 protein is identified, biochemical analysis, namely measuring the subunit stoichiometry and affinity to Gβ, canonical Gα, and XLGs, is possible. Although we tried to identify the native Gγ3 protein using an anti-Gγ3 domain antibody, the antibody recognized multiple proteins. To identify the native Gγ3 protein, we use the *RGG3* mutants *MINUTE (Mi)* and *GS3-3*, which produce partially defective proteins, as references for subtraction to Taichung 65 (abbreviated as WT [wild-type] hereinafter). Here, we find a candidate of the native RGG3 protein, Gγ3. Finally, we confirmed that the candidate was the native Gγ3 protein using nano-liquid chromatography-tandem mass spectrometry (LC-MS/MS) analysis of the immunoprecipitation products using an anti-Gγ3 domain antibody. Using this antibody, the subcellular localization and tissue-specific accumulation of the native Gγ3 protein were studied.

## 2. Result

### 2.1. Morphology of Rice Heterotrimeric G Protein γ3 Gene (RGG3/GS3/Mi/OsGGC1) Mutants:

**Figure 1:**
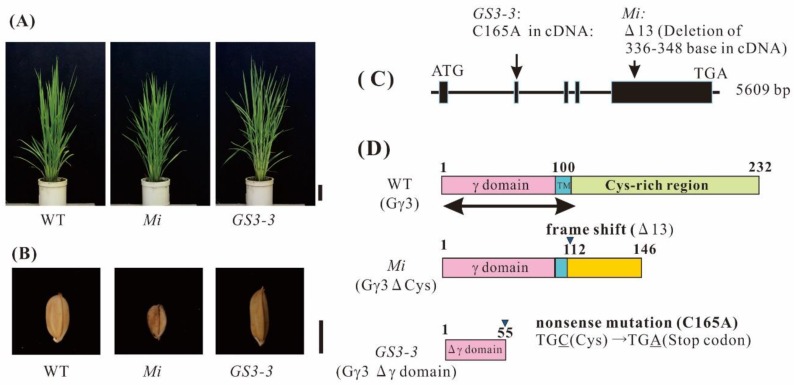
Morphology of rice heterotrimeric G protein γ3 gene (*RGG3/GS3/Mi/OsGGC1*) mutants, and genome and protein structure of *RGG3/GS3/Mi/OsGGC1*. (**A**) Gross morphology of the wild-type (WT) (Taichung 65), *Mi* and *GS3-3*; Bar = 10 cm. (**B**) Seed morphologies of the plants in (**A**); Bar = 5 mm. (**C**) Genome structure of *RGG3/GS3/Mi/OsGGC1* and positions of mutations in *RGG3/GS3/Mi/OsGGC1* mutants, *Mi* and *GS3-3*. The 13-base deletion (336–348th base in full-length cDNA) and one base substitution (C165A in full-length cDNA) had occurred in *Mi* and *GS3-3*, respectively. In *GS3-3*, a codon, TGC (cysteine) changed to TGA (stop codon). (**D**) Protein structure of the product of *RGG3/GS3/Mi/OsGGC1* in the WT (Gγ3), *Mi* (Gγ3ΔCys), and *GS3-3* (Gγ3Δγ domain). The canonical γ domain region is shown as γ domain (pink bar). The putative transmembrane domain is indicated as TM (blue bar). The region with cysteine abundance is labeled as cysteine-rich region (green bar). The newly produced amino acid sequence by the frame shift resulting from of 13-base deletion is indicated with a yellow bar. An arrow under the WT Gγ3, which covers 120 amino acid residues from N-terminal, is the region used for recombinant proteins, such as the thioredoxin (Trx)-tagged Gγ3 domain protein (Trx-Gγ3 domain protein), used as an antigen, and the glutathione S transferase (GST)-tagged Gγ3 domain protein (GST-Gγ3 domain protein), used for affinity purification of the antibody.

To confirm the functions of rice heterotrimeric G protein γ3 subunit in determining the plant morphology, we prepared plants possessing *GS3-3* [30] and *Mi* [58] mutation with Taichung 65 as a background. The mutant, *Mi* was slightly dwarfed (Figure 1A) and set small seeds (Figure 1B), compared to those of the WT. *GS3-3* had a height similar to that of the WT (Figure 1A) and set large seeds (Figure 1B). These results indicate that the mutations in *Mi* and *GS3-3* clearly affected the seed size.

### 2.2. Genomic Structure of RGG3 and Protein Structure of Gγ3

The genome sequence of *RGG3* was found in RAP-DB (Os03g0407400). We reconfirmed the genome sequence of *RGG3. RGG3* consists of five exons (Figure 1C) and its translation product, Gγ3, comprises 232 amino acid residues. In order to prepare recombinant proteins, cDNA for RGG3 was isolated. The molecular weight of Gγ3 calculated from the cDNA, was 24249 Da. The Gγ3 consists of the canonical γ domain (about 100 amino acid residues), a short region with hydrophobic amino acid residues (tentatively named transmembrane region: TM), and a region with a large number of cysteines (Cys-rich region) (Figure 1D).

The *Mi* mutation occurred as a result of the deletion of 13 bases in *RGG3*. The mutation site corresponds to 336–348th positions in the full-length cDNA of *RGG3*, resulting in a frame-shift (Figure 1C). We reconfirmed the mutation in *Mi*. In *Mi,* the mutated protein, tentatively named Gγ3∆Cys, consists of 146 amino acid residues (Figure 1D). The cysteine-rich region is absent in Gγ3∆Cys. The molecular weight of Gγ3∆Cys, calculated from cDNA, was 15,651 Da.

The *GS3-3* mutation occurred as a result of one base substitution. The C at the 165th position in the full-length cDNA of *RGG3* was substituted by A (C165A), resulting in the generation of a stop codon (Figure 1C). As the mutation in TCM3-467 was the same as that in *GS3-3* [18], we renamed TCM3-467 to *GS3-3*. The *GS3-3* mutation generated a mutated protein with 55 amino acid residues, tentatively named the Gγ3∆γ domain (Figure 1D). The Gγ3∆γ domain is an immature protein lacking about half of the canonical γ domain. The molecular weight of the Gγ3∆γ domain, calculated from cDNA, was 5653 Da. The chemiluminescent intensity of Gγ3ΔCys was more than 7-fold that of Gγ3, when 10 μg of protein of the plasma membranes of the WT and *Mi*, respectively, was analyzed by western blot.

### 2.3. Gγ3 Candidates Localized in the Plasma Membrane Fraction

**Figure 2:**
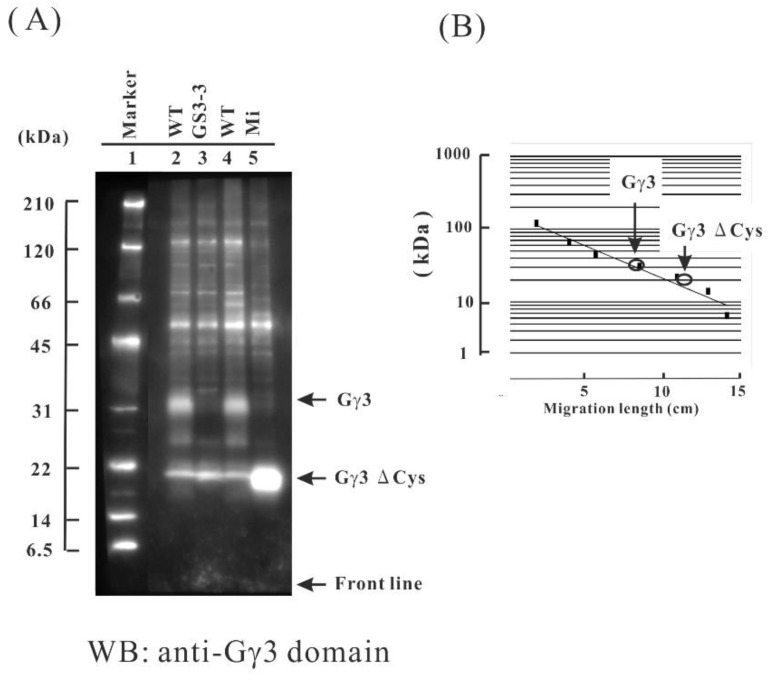
Immunological study of the Gγ3 candidates in the wild-type (WT), *Minute* (*Mi*), and *GS3-3* flowers. (**A**) First, 10 μg of each protein of the plasma membrane fractions of the WT and *GS3-3* and 5 μg of the protein of the plasma membrane fractions of *Mi* were used for the Western blot analysis using an anti-Gγ3 domain antibody. Molecular weight marker (lane 1). The Gγ3 candidate was detected as a broad band with a molecular weight of approximately 32 kDa in the WT (lanes 2 and 4). No Gγ3 was detected in *GS3-3* (lane 3). The Gγ3ΔCys candidate was detected as a band with a molecular weight of approximately 20 kDa in *Mi* (lane 5). (**B**) The molecular weights of Gγ3 and Gγ3ΔCys candidates were estimated using a molecular weight marker as a standard.

Identification of native Gγ3 was carried out by Western blotting. As mutants have no native full length Gγ3, these were used as references, in order to identify native Gγ3 in WT. The plasma membrane fraction was chosen in this study as it was shown that Gα and Gβ accumulated in plasma membrane fraction in rice. The plasma membrane fractions of WT, *GS3-3*, and *Mi* flowers were prepared using an aqueous two-polymer phase system, and Gγ3 candidates were detected by Western blotting using an anti-Gγ3 domain antibody. In the WT, a 32-kDa protein (Gγ3 candidate) was detected (Figure 2A, lanes 2 and 4); this band was not observed in *GS3-3* or *Mi*. The molecular weight of the Gγ3 candidate is much higher than that of Gγ3 calculated from the cDNA of the WT (24 kDa). In *GS3-3*, the Gγ3∆γ domain was not detected (Figure 2A, lane 3). In *Mi*, a 20-kDa protein (Gγ3∆Cys candidate) was detected (Figure 2A, lane 5). The molecular weight of the Gγ3∆Cys candidate was much higher than that of Gγ3∆Cys calculated from the cDNA (16 kDa). The molecular weights of Gγ3 and Gγ3∆Cys candidates were measured using molecular weight markers (Figure 2B).

### 2.4. Immunoprecipitation of Gγ3 and Gγ3∆Cys Using an Anti-Gγ3 Domain Antibody

**Figure 3:**
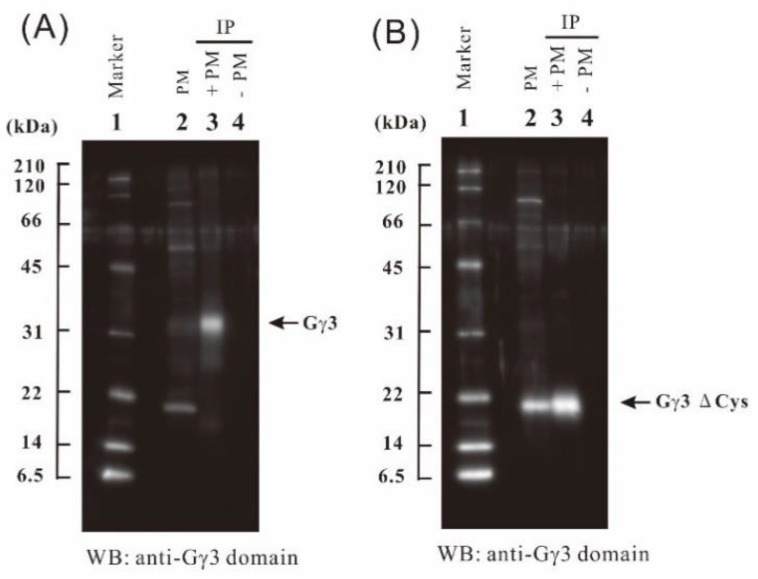
Immunoprecipitation of Gγ3 and Gγ3ΔCys candidates. (**A**) Immunoprecipitation of the Gγ3 candidate from solubilized plasma membrane proteins of the wild-type (WT) flower using an anti-Gγ3 domain antibody. Molecular weight marker (lane 1); 10 μg of protein of the plasma membrane fraction of the WT (lane 2); the immunoprecipitation product of solubilized plasma membrane proteins and anti-Gγ3 domain antibody (lane 3); control experiment (buffer in place of the membrane protein; lane 4). (**B**) Immunoprecipitation of the Gγ3ΔCys candidate from the solubilized plasma membrane proteins of the *Minute* (*Mi*) flower using an anti-Gγ3 domain antibody. Molecular weight marker (lane 1); 10 μg of protein of the plasma membrane fraction of *Mi* (lane 2); the immunoprecipitation product of the solubilized plasma membrane proteins of *Mi* and the anti-Gγ3 domain antibody (lane 3); control experiment (buffer in place of the membrane protein; lane 4).

To concentrate Gγ3 and Gγ3∆Cys candidates, immunoprecipitation was carried out using anti-Gγ3 domain antibody. First, 50 μg of the anti-Gγ3 domain antibody was added to 1 mg each of solubilized plasma membrane protein of the WT (Figure 3A) and *Mi* (Figure 3B) flowers. Gγ3 and Gγ3∆Cys candidates were collected with the antibody cross-linked Protein A bound beads. The 32 kDa protein, a Gγ3 candidate in the WT (Figure 3A, lane 3) and 20-kDa protein, a Gγ3∆Cys candidate in *Mi* (Figure 3B, lane 3), were immunoprecipitated.

### 2.5. LC-MS/MS Analysis

To demonstrate that Gγ3 and Gγ3ΔCys candidates are actually Gγ3 and Gγ3ΔCys, and that proteins with which the anti-Gγ3 domain antibody reacted, are actually Gγ3 and Gγ3ΔCys, LC-MS/MS analysis was carried out. First, using LC-MS/MS, we checked for Gγ3 and Gγ3ΔCys candidates in the eluate from the gel containing plasma membrane proteins following SDS-PAGE. When the signal intensities of Gγ3 and Gγ3ΔCys candidates detected by LC-MS/MS were not enough, we analyzed immunoprecipitation products, enriched with anti-Gγ3 domain antibody.

**Figure 4:**
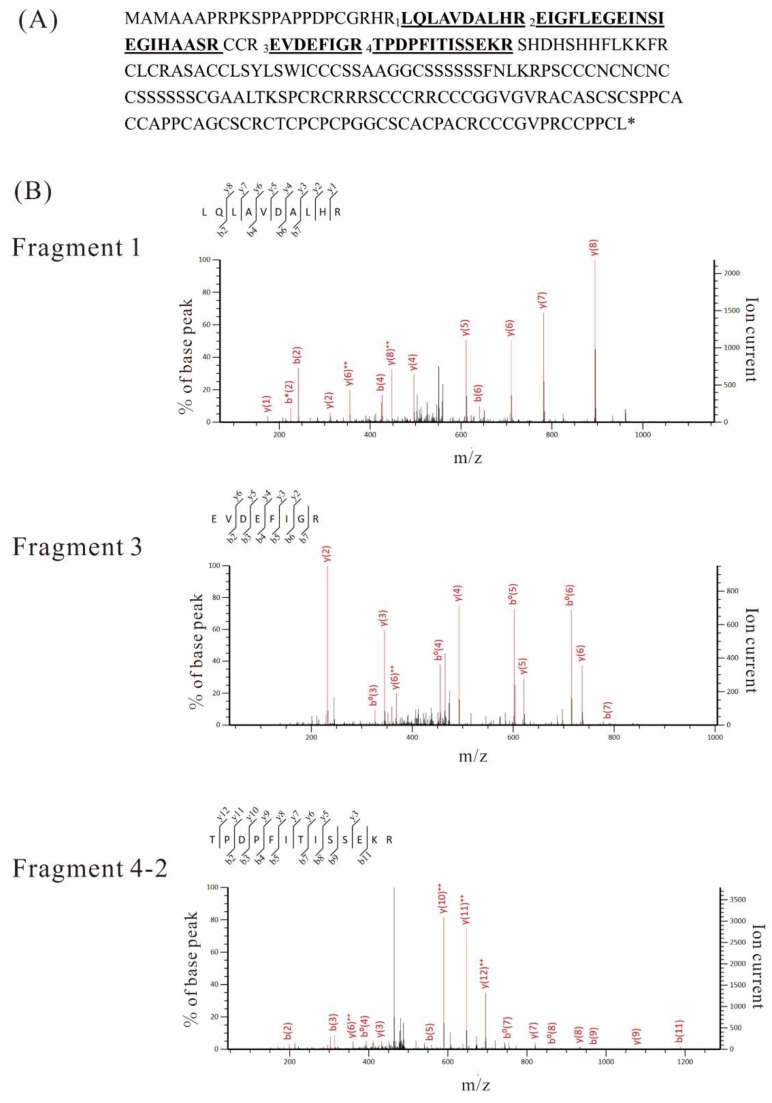
LC-MS/MS analysis of Gγ3 candidates. (**A**) Four peptides (*p* < 0.05), which were produced by trypsin-digested Gγ3 candidates in the wild-type (WT) and *Mi*, were numbered and underlined in the full length Gγ3 amino acid sequence. These peptides are listed in Table 1. (**B**) MS/MS spectra of the three fragments, which were obtained from the immunoprecipitation product of Gγ3 in the WT (Figure 3A, lane 3). Fragment numbers correspond to Table 1C.

First, plasma membrane proteins from the WT and *Mi* were analyzed by LC-MS/MS. 40 μg of each flower plasma membrane protein from WT and *Mi* was separated by SDS-PAGE and each lane was separated into 10 pieces to increase the relative amount of target proteins, according to the molecular weight marker. After these gel pieces were digested with trypsin, peptides were analyzed by LC-MS/MS in triplicate. Typical examples are summarized in Table 1. Fragments were assigned to the sequence of Gγ3, and their positions are indicated in Figure 4A.

In the analysis of the plasma membrane fraction of the WT, three Gγ3 fragments (fragments 1, 2, and 3) (*p* < 0.05) were detected in a gel piece containing a 32 kDa protein (Table 1A). In the plasma membrane fraction of *Mi*, three Gγ3 fragments (fragments 2, 3, and 4-1) (*p* < 0.05) were detected in a gel piece containing a 20 kDa protein (Table 1B).

Immunoprecipitation products were separated by SDS-PAGE and analyzed by LC-MS/MS. Immunoprecipitation products from the WT and *Mi* were not detected by silver staining (data not shown). In the immunoprecipitation products of the WT (Figure 3A, lane 3), a gel piece containing a 32 kDa protein was cut and digested with trypsin, and the resultant peptides were analyzed by LC-MS/MS. As a result, three Gγ3 fragments (fragments 1, 3, and 4-2), represented as primary mass (*p* < 0.05), were obtained (Table 1C). Fragment 4-2 is an incomplete trypsin-digested fragment containing an arginine residue (R) at its C-terminus, making it differ from fragment 4-1. In the immunoprecipitation products of *Mi*, a gel piece containing a 20-kDa protein was cut and digested by trypsin, and the resultant peptides were analyzed by LC-MS/MS. As a result, four fragments (fragments 1, 2, 3, and 4-1) (*p* < 0.05) were obtained (Table 1D).

The MS/MS results of fragments 1, 3, and 4-2 are shown in Figure 4B. Based on these results, we concluded that the 32 kDa and 20 kDa polypeptides were Gγ3 and Gγ3∆Cys, respectively. When the immunoprecipitation product of the WT was analyzed by LC-MS/MS, five fragments, SPCRCR, SCCCRR, RCCCGGVGVR, ACASCSCSPPCACCAPPCAGCSCR, and CCPPCL, which were positioned at the C-terminal parts of Gγ3, were detected by the Mascot search, but their scores were very low (Mascot score < 11). Therefore, these five fragments were excluded from Table 1 and Figure 4A.

When the NCBI protein database was used for the analysis of Gγ3 candidates, Gγ3 was annotated using another name, BAH89202.1

### 2.6. Gγ3 and Gγ3∆Cys Were Enriched in the Plasma Membrane Fraction

**Figure 5:**
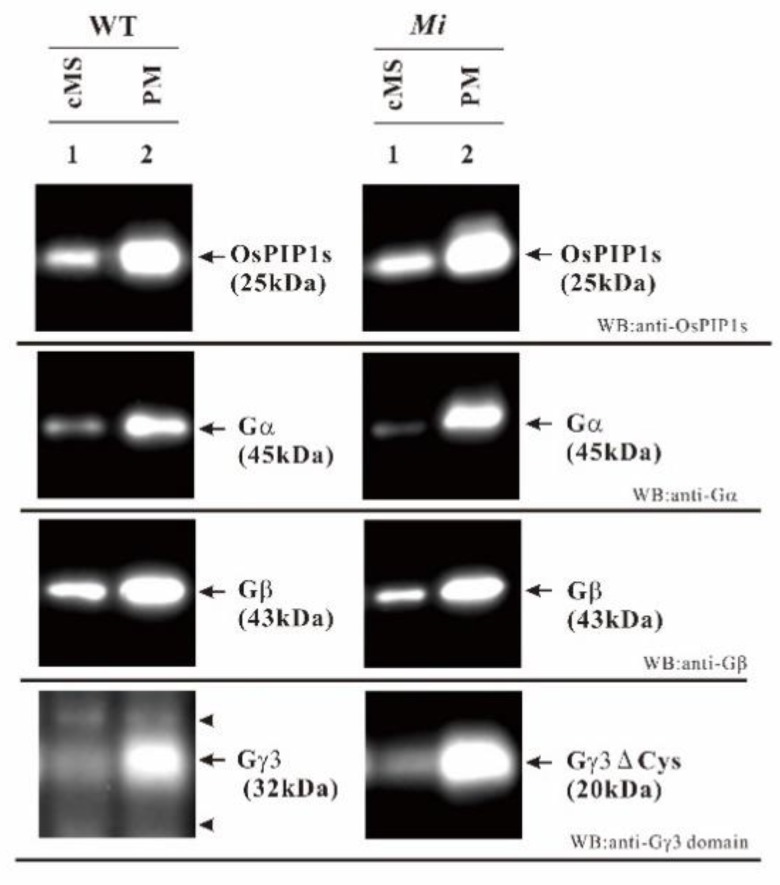
Gγ3 and Gγ3ΔCys were enriched in the plasma membrane fraction of the wild-type (WT) and *Minute* (*Mi*) flowers. First 10 μg of both the crude microsomal fraction protein and plasma membrane fraction protein from the WT and *Mi* were analyzed by western blot using anti-OsPIP1s, anti-Gα, anti-Gβ, and anti-Gγ3 domain antibodies. OsPIP1s is an aquaporin, which is a plasma membrane marker. OsPIP1s (25 kDa), Gα (45 kDa), Gβ (4 3kDa), Gγ3 (32 kDa), and Gγ3ΔCys (20 kDa) are indicated by arrows. Non-specific bands are indicated by arrow heads.

To check whether Gγ3 and Gγ3∆Cys are enriched in the plasma membrane, the amount of Gγ3 and Gγ3∆Cys in the crude microsomal fraction was compared with that in the plasma membrane fraction (Figure 5). Tissue-homogenate was centrifuged at 10,000× *g* for 10 min and the resulting supernatant was centrifuged at 100,000× *g* for 1 h. The precipitate (100,000 g ppt) was named the crude microsomal fraction (cMS). The plasma membrane fractions were prepared from cMS, using the aqueous two-polymer phase system.

OsPIP1s is an aquaporin, which is a plasma membrane marker. Gα and Gβ are the subunits of the heterotrimeric G protein complex in rice. The OsPIP1s, Gα subunit, and Gβ subunit were enriched in the plasma membrane fraction. Furthermore, Gγ3 (32 kDa in WT) and Gγ3∆Cys (20 kDa in *Mi*) were also enriched in the plasma membrane fraction. These results showed that Gγ3 (32 kDa in WT) and Gγ3∆Cys (20 kDa in *Mi*) were localized in the plasma membrane fraction.

### 2.7. Tissue-Specific Accumulation of Gγ3

**Figure 6:**
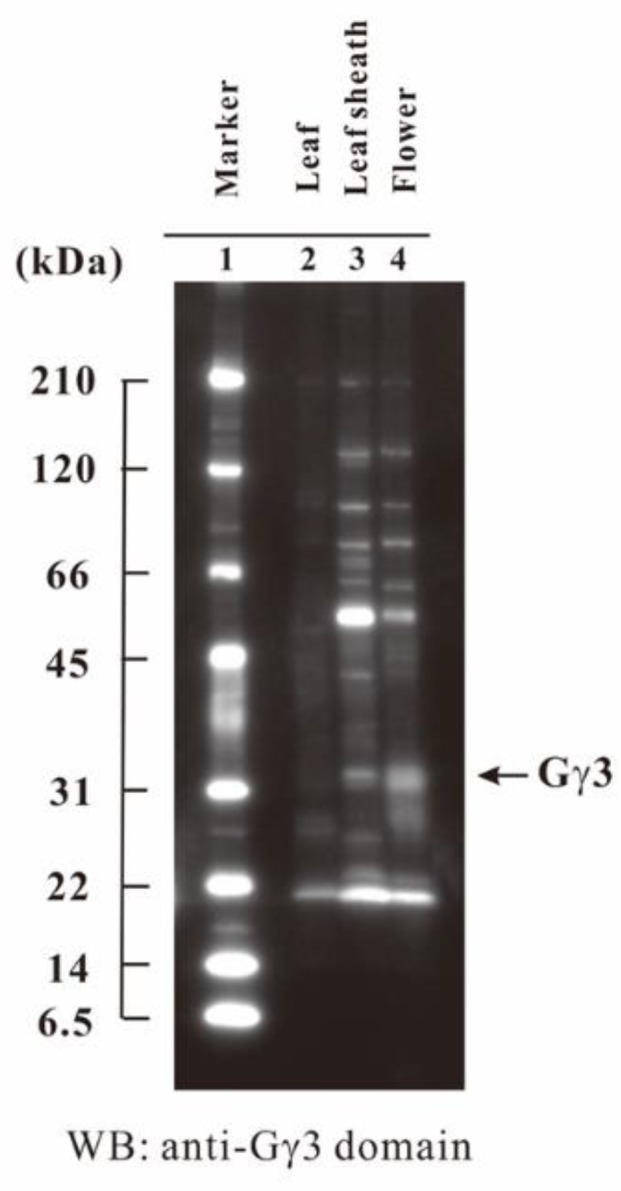
Tissue-specific accumulation of Gγ3 in the wild-type (WT). Ten micrograms of each of the plasma membrane fraction proteins of the leaf, leaf sheath, and flower in the WT was analyzed by SDS-PAGE and Western blotting using an anti-Gγ3 domain antibody. Molecular weight marker (lane 1); leaf from etiolated seedling (lane 2); developing leaf sheath at the eighth leaf stage (lane 3); 1–5 cm flower (lane 4).

In order to know the tissues in which Gγ3 accumulates, the accumulation profile of Gγ3 was studied using the plasma membrane fractions of one-week-old etiolated seedlings of WT, developing leaf sheaths, and flowers. The results showed that the Gγ3 protein largely accumulated in the developing flower (Figure 6).

## 3. Discussion

In rice, there are three atypical γ subunit genes (*RGG3*, *RGG4*, and *RGG5*) that are homologous to *AGG3*. The tentatively named *RGG3* corresponds to *GRAIN SIZE 3 (GS3),* which is a gene that regulates seed length [19,30,56,57,58] and *RGG4* corresponds to *DENSE AND ERECT PANICLE1* (*DEP1*), which is a gene that regulates plant architecture including semi-dwarfness, panicle number and panicle erectness [20,55]. *RGG5* corresponds to *GGC2* [21], which a gene that increases grain length in combination or individually with *DEP1* [56]. These genes are important for rice breeding. These have been already cloned, but their native translation products have not yet been studied. In this study, we focused on the native translation products of *RGG3/GS3/Mi/OsGGC1*.

First, we detected the Gγ3 candidate from the WT and the truncated Gγ3 candidate (Gγ3∆Cys) from *Mi* by Western blotting using anti-Gγ3 domain antibodies (Figure 2A). In SDS-PAGE, the molecular weights of the Gγ3 and Gγ3∆Cys candidates were estimated as 32 and 20 kDa, respectively, which were larger than the molecular mass calculated using cDNAs, i.e., 24 and 16 kDa, respectively. These results indicate that modifications, such as glycosylation, ubiquitination, phosphorylation, and lipid modification (palmitoylation etc.), may have occurred after translation in the Gγ3 and Gγ3∆Cys candidates. The identification of the modification is a subject requiring further study. In order to obtain concrete evidence on whether the Gγ3 and Gγ3∆Cys candidates detected by western blotting were actually Gγ3 and Gγ3∆Cys proteins, the immunoprecipitation products of the Gγ3 and Gγ3∆Cys candidates were analyzed by LC-MS/MS (Figure 3 and Figure 4). As a result, four fragments, with *p* < 0.05 by the Mascot search engine, were obtained from the Gγ3 and Gγ3∆Cys candidates. These results indicated that the Gγ3 and Gγ3∆Cys candidates were actually Gγ3 and Gγ3∆Cys, respectively.

Mutants of *RGG3*, i.e., *Mi* [58] and *GS3-3* [30], set small and large seeds, respectively (Figure 1B). Thus, *RGG3* regulates seed morphology. Gγ3 and Gγ3∆Cys were accumulated in the plasma membrane fraction of the flower tissue (Figure 5). The tissue in which Gγ3 and Gγ3∆Cys were accumulated corresponded to the tissue that showed the morphological abnormalities in *Mi* and *GS3-3* (Figure 1 and Figure 6). One of the deletion alleles of *GS3* decreased the cell number in the lemma and palea and a knock-down construct of *GS3* utilizing RNAi increased the cell number [58]. Gγ3 also modulates cell proliferation, similar to Gα [31] and Gβ [32]. The chemiluminescent intensity of Gγ3ΔCys was more than 7-fold that of Gγ3 (Figure 2). The reason that the amount of Gγ3 was fewer than that of Gγ3ΔCys may be that Gγ3 is degraded by proteases. Another possibility could be that Gγ3ΔCys may stably accumulate in the plasma membrane with other proteins, including Gβ. Hence, further analysis of native and truncated Gγ3s will be important to understanding seed size regulation.

Sun et al. reported that GS3-1 (corresponding to Gγ3) interacted with Gβ using a Y2H assay [56]. Using BiFC, they also revealed that GS3-1 and GS3-4, truncated Gγ3 proteins in *GS3-4*, interacted with Gβ on the plasma membrane [56]. GS3-4 in *GS3-4* [30,56] and Gγ3∆Cys in *Mi* [58] consisted of 149 and 146 amino acid residues, respectively. GS3-4 and Gγ3∆Cys have the canonical Gγ domain and a putative transmembrane domain, but largely lack a cysteine-rich domain. In this study, native Gγ3 and Gγ3∆Cys were enriched in the rice plasma membrane, similar to the Gβ subunit (Figure 5). We also confirmed that Gγ3 and Gγ3∆Cys interacted with Gβ using a Y2H assay (data not shown). From these results, it is suggested that Gγ3 and Gγ3∆Cys may form a dimer with Gβ on the plasma membrane. As we identified Gγ3 and Gγ3∆Cys by immunological techniques and LC-MS/MS analysis in this study, it will be possible to research whether the Gγ3 protein is a component of the heterotrimeric G protein complex containing the canonical Gα and XLGs.

As the seeds of *Mi* [58] and *GS3-4* [30,56] were shorter than those of the WT, Gγ3∆Cys is the cause of shortened seeds. It will be important to clarify whether Gγ3∆Cys interacts with Gβ. If the βγ dimer composed with Gγ3∆Cys is present in the plasma membrane, it will be interesting to research the interaction between the unusual βγ dimer (GβGγ3∆Cys) and the canonical Gα or XLGs, on the basis of the G protein signaling model [5,6]. As previously reported, some βγ dimers seem to be present in two different fractions in gel filtration: one is a component of a huge complex, and the other is a sole βγ dimer in the plasma membrane of etiolated rice seedlings [18]. Although this may be the result of artificial dissociation during solubilization and gel fractionation, this approach will be important for understanding the heterotrimeric G protein complex. Truncated Gγ3 in *GS3-3*, namely the Gγ3∆γ domain, consisted of 55 amino acid residues, which is considered as a loss of function of OSR (organ size regulation) [30]. In *GS3-3*, the Gγ3∆γ domain was not detected in the plasma membrane (Figure 2A). The reason may be due to the lack of the trans-membrane domain in the Gγ3∆γ domain or due to the lack of sites that anti-Gγ3 domain antibody recognizes in the Gγ3∆γ domain. In addition, the Gγ3∆γ domain was not detected in the cytosolic fraction (data not shown). However, it is not ruled out that there is no Gγ3∆γ domain in the cytosolic fraction, due to the detection threshold in Western blot not being met. As seeds of *GS3-3* were longer than those of the WT, the lack of a βγ3 dimer may be the cause of enlarged seeds. Hence, as we detected Gγ3 and Gγ3∆Cys proteins in this study, biochemical analysis of the heterotrimeric G protein complex in *Mi* and *GS3-3* will be accelerated. It is of interest to reveal the subunit stoichiometry of the canonical Gα, XLGs, Gβ, and five Gγs, namely γ1, γ2, the Gγ3∆γ domain, γ4, and γ5, and the subsequent subunit composition of the G protein complex in *Mi*, which sets small grains. It is also important to analyze the subunit stoichiometry of the canonical Gα, XLGs, Gβ, and four Gγs, namely γ1, γ2, γ4, and γ5, and the subsequent subunit composition of the G protein complex in *GS3-3*, which sets large grains.

## 4. Materials and Methods

### 4.1. Plant Materials

A rice cultivar (*Oryza sativa* L. cv. Taichung 65) and two heterotrimeric G protein γ3 mutants (*GS3-3* and *Mi*) were used in this study. *GS3-3* was obtained from the Taichung 65 mutant library, mutagenized by N-methyl-N-nitrosourea treatment, and named TCM-3-467. The *Mi* mutation was provided from the stocked mutant line, H343 (*Oryza sativa* L. cv. Akamuro background). H343 was backcrossed four times with Taichung 65, and was used as a near-isogenic line of *Mi* in this study. All rice plants were grown under a 14-h light (50,000 lux and 28 °C) and 10-h dark (25 °C) cycle, or under natural field conditions.

### 4.2. Sequencing and Confirmation of RGG3

Genomic DNA was isolated from whole plants of WT, *Mi,* and *GS3-3* using an extraction method with cetyltrimethylammonium bromide (CTAB) [59]. Using this as a template, PCR was performed using > 20 sets of PCR primers to cover 5609 bases of *RGG3* (Os03g0407400). The amplified DNA fragments were directly sequenced using the same primers that were used for amplification.

### 4.3. RNA Isolation, Reverse Transcription, and cDNA Encoding of the Heterotrimeric G Protein Gγ3 Subunit

Total RNA from the flower tissue was directly extracted using RNeasy Plant Mini kits (Qiagen, Hilden, Germany). The first strand of cDNA was synthesized using Super Script First Strand Synthesis System for RT-PCR (Invitrogen, Carlsbad, CA, USA). Total RNA (0.5 μg) and oligo-dT were used as the template and primer, respectively, for the first strand cDNA synthesis.

In order to isolate *RGG3* cDNA, the primers were designed based on the database information (Os03g0407400):

RGG3 forward: 5’ atggcaatggcggcggcgcc 3’;
RGG3 reverse: 5’ caagcagggggggcagcaac 3’.

The amplified PCR products were sub-cloned into pCR4 (Invitrogen) and sequenced with a Thermo BigDye Terminator Cycle Sequencing Kit (Amersham Biosciences, Little Chalfont, UK) using a DNA sequencer (Model 377; Applied Biosystems, Foster City, CA, USA).

### 4.4. Preparation of the Microsomal and Plasma Membrane Fractions in Rice

Crude microsomal fractions were prepared from 2–5 cm flowers of the WT, *Mi,* and *GS3-3*, as described previously [18], and plasma membrane fractions were purified from the crude microsomal fraction using an aqueous two-polymer phase system [60]. From the etiolated seedlings, which were grown for 5 d at 28 °C, and developing leaf sheaths at the eighth leaf stage, crude microsomal fractions and plasma membrane fractions were prepared, respectively.

### 4.5. SDS-Polyacrylamide Gel Electrophoresis (SDS-PAGE)

Electrophoresis was carried out on 12.5% and 10/20% gradient polyacrylamide gels containing 0.1% SDS, as described previously [61].

For LC-MS/MS analysis, 40 μg of flower plasma membrane proteins from both the WT and *Mi* were analyzed using 15% SDS-PAGE. Electrophoresis was stopped at a position where the Bromophenol Blue was 3 cm away from the stacking gel. The 3-cm long gel was divided into 10 pieces according to the molecular weight marker (Precision Plus Protein^TM^ Kaleidoscope^TM^; Bio-Rad Laboratories), without staining. These gel pieces were used for trypsin digestion. In some cases, gels were silver-stained using Pierce Silver Stain for Mass Spectrometry (Thermo Scientific).

### 4.6. Preparation of Trx-Gγ3 and GST-Gγ3 Domain Proteins

cDNA encoding 120 amino acid residues from the N-terminal of the rice Gγ3 protein was amplified by PCR using primers. The cDNA contains the Gγ3 domain and the putative transmembrane region:

RGG3 domain forward: 5’ccttggctcatatggatatcatggcaatggcggcggcgccccggcccaag3’;
RGG3 domain reverse: 5’aagcttcccgggtcaggaggaggatgagcagccgccggcggcgctgctg3’.

Amplified cDNA was sub-cloned in pET32a containing thioredoxin (Trx) and histidine (His) tags (Novagen). The resultant clone, the Trx-Gγ3 domain vector, was transformed in T7 Express *lysY*/*I^q^ E. coli* (New England Biolabs), and the recombinant protein was synthesized and designated as the Trx-Gγ3 domain protein. The cDNA covering the Gγ3 domain was also sub-cloned in pET41 containing glutathione S-transferase (GST) and His tags (Novagen). The resultant clone, the GST-Gγ3 domain vector, was transformed in T7 Express *lysY/I^q^ E. coli* (New England Biolabs), and the recombinant protein was synthesized and designated as the GST-Gγ3 domain protein.

The overexpression of the Trx-Gγ3 domain protein and GST-Gγ3 domain protein in T7 Express *lysY/I^q^ E. coli* was carried out as described elsewhere [61]. Inductions were performed at 37 °C. Induction was initiated by the addition of IPTG (final IPTG concentration, 1 mM). After 3 h, *E. coli* was harvested after centrifugation at 10,000 × *g* for 5 min at 4 °C, and stocked at −80 °C before use.

As the Trx-Gγ3 domain protein and GST-Gγ3 domain protein were included in the body, both proteins were solubilized in 6 M guanidine hydrochloride, 10 mM Tris HCl, pH 8.0. Solubilized proteins were applied to Ni-NTA agarose (Qiagen, Hilden, Germany). The purification of both proteins was performed according to the protocols recommended by the manufacturers.

The antibody was raised against the Trx-Gγ3 domain protein in rabbits. Affinity purification of the antibody was performed using a polyvinylidene fluoride (PVDF) filter (Millipore, Burlington, MA, USA), immobilized with the GST-Gγ3 domain protein.

### 4.7. Western Blot Analysis (WB)

Proteins were separated by 12.5% or 10/20% gradient SDS-PAGE, and blotted onto a PVDF membrane (Millipore). The antibody against the rice Gγ3 domain was affinity-purified in this study. Antibodies against the rice heterotrimeric G protein α and β subunits, namely the anti-Gα and anti-Gβ antibodies, were used as described previously [18]. The antibody against aquaporin (a plasma membrane marker), namely, anti-OsPIP1s, was purchased from Operon Biotechnologies. The Chemi-Lumi One Markers Kit (Nacalai Tesque, Kyoto, Japan) was used as a molecular weight marker for western blotting.

ECL^TM^ peroxidase labelled anti-rabbit antibody was purchased as second antibody from GE Healthcare, Little Chalfont, UK. ECL Immobilon^TM^ Western Chemiluminescent HRP Substrate (Millipore, Burlington, MA, USA) was used as the western blotting detection reagent. The chemiluminescent signal was measured using a Fusion SL (MS instruments).

### 4.8. Immunoprecipitation

First, 50 μg of affinity-purified anti-Gγ3 domain antibody was bound to 50 mg of Protein A bound magnetic beads (Millipore, Burlington, MA, USA). After washing them thrice with 1× PBS, the anti-Gγ3 domain antibody and Protein A were cross-linked with dimethyl pimelimidate dihydrochloride (DMP). The conditions followed for cross-linking were according to the protocols recommended by the manufacturers. After quenching the magnetic cross-linked beads with the anti-Gγ3 domain antibody, they were stored at 4 °C until use.

Next, 0.1 mL of 10% SDS was added to 0.9 mL of plasma membrane fraction (1 mg protein/10 mg SDS/mL) and denatured for 5 min at 90 °C. After diluting the solubilized fraction with 10 mL of 1× TBS containing 1% Tween 20, the magnetic beads cross-linked with 50 μg of the anti-Gγ3 domain antibody were added. After incubation for 2 h at 25 °C, the magnetic beads were collected into a 1.5 mL tube and washed thrice each with 0.5 mL of 1× TBS containing 0.1% Tween 20 and 0.5 mL of 1× TBS. Proteins were eluted using 40 μL of dissociation buffer (Bio-rad) without a reducing agent, from the beads. In total, 5 μL of each eluate was used for LC-MS/MS.

### 4.9. Protein Reduction, Alkylation, and Trypsin Digestion for LC-MS/MS Analysis

Gel pieces were resuspended in 50 mM NH_4_HCO_3_, reduced with 50 mM dithiothreitol for 30 min at 56 °C, and alkylated with 50 mM iodoacetamide for 30 min at 37 °C in the dark. Alkylated proteins in the gels were digested with 10 μg/mL of trypsin solution (Promega, Madison, WI, USA) for 16 h at 37 °C. The resultant peptides were concentrated and suspended in 0.1% formic acid and analyzed by LC-MS/MS.

### 4.10. Protein Identification Using Nano-LC-MS/MS

The peptides were loaded onto the LC system (EASY-nLC 1000; Thermo Fisher Scientific, Waltham, MA, USA) equipped with a trap column (EASY-Column, C18-A1 5 µm, 100 µm ID × 20 mm; Thermo Fisher Scientific), equilibrated with 0.1% formic acid, and eluted with a linear acetonitrile gradient (0–50%) in 0.1% formic acid at a flow rate of 200 nL/min. The eluted peptides were loaded and separated on the column (C18 capillary tip column, 75 µm ID × 120 mm; Nikkyo Technos, Tokyo, Japan) with a spray voltage of 1.5 kV. The peptide ions were detected using MS (LTQ Orbitrap Elite MS; Thermo Fisher Scientific) in the data-dependent acquisition mode with Xcalibur software (version 2.2; Thermo Fisher Scientific). Full-scan mass spectra were acquired in MS over 400–1500 *m*/*z* with a resolution of 60,000. The 10 most intense precursor ions were selected for collision-induced fragmentation in the linear ion trap, at a normalized collision energy of 35%. Dynamic exclusion was employed within 90 s to prevent the repetitive selection of peptides.

### 4.11. MS Data Analysis

Protein identification was performed using the Mascot search engine (version 2.5.1, Matrix Science, London, UK) and the in-house database, which constructed the amino acid sequences of rice heterotrimeric G protein subunits. For both the searches, the carbamidomethylation of cysteine was set as a fixed modification, and oxidation of methionine was set as a variable modification. Trypsin was specified as the proteolytic enzyme and one missed cleavage was allowed. The peptide mass tolerance was set at 10 ppm, fragment mass tolerance was set at 0.8 Da, and peptide charges were set at +2, +3, and +4. An automatic decoy database search was performed as part of the search. Mascot results were filtered with the Percolator function to improve the accuracy and sensitivity of peptide identification. The minimum requirement for the identification of a protein was two matched peptides. Significant changes in the abundance of proteins between samples were determined (*p* < 0.05).

### 4.12. Gene ID

The accession numbers of the rice heterotrimeric G proteins α, β, and γ3 subunit genes (*RGA1*, *RGB1*, and *RGG3*, respectively) are Os05g0333200, Os03g0669200, and Os03g0407400, respectively.

## Figures and Tables

**Table 1:** LC-MS/MS analysis of Gγ3 fragments in in the plasma membrane of the wild-type (WT) and *Minute* (*Mi*) flowers.

**(A)** Gγ3 fragments in the plasma membrane fraction of the WT flower					
**Fragments**	**Observed**	**Mr(expt)**	**Mr(calc)**	**Expected**	**Peptide**
1	379.2251	1134.6536	1134.6509	0.00078	R.LQLAVDALHR.E
2	714.7006	2141.08	2141.0753	0.00000034	R.EIGFLEGEINSIEGIHAASR.C
3	482.7414	963.4682	963.4662	0.007	R.EVDEFIGR.T
(**B**) Gγ3 fragments in the plasma membrane fraction of the *Mi* flower					
**Fragment**	**Observed**	**Mr(expt)**	**Mr(calc)**	**Expected**	**Peptide**
2	714.7014	2141.0824	2141.0753	0.0000024	R.EIGFLEGEINSIEGIHAASR.C
3	482.7408	963.4671	963.4662	0.0077	R.EVDEFIGR.T
4-1	667.8468	1333.6791	1333.6765	0.00015	R.TPDPFITISSEK.R
(**C**) Gγ3 fragments in the immunoprecipitation products using the plasma membrane fraction of WT flower					
**Fragments**	**Observed**	**Mr(expt)**	**Mr(calc)**	**Expected**	**Peptide**
1	568.3348	1134.6551	1134.6509	5.40 × 10^−7^	R.LQLAVDALHR.E
3	482.7417	963.4688	963.4662	0.00062	R.EVDEFIGR.T
4-2	497.6015	1489.7827	1489.7776	2.50 × 10^−5^	R.TPDPFITISSEKR.S
(**D**) Gγ3 fragments in the immunoprecipitation products using the plasma membrane fraction of *Mi* flower					
**Fragments**	**Observed**	**Mr(expt)**	**Mr(calc)**	**Expected**	**Peptide**
1	568.3354	1134.6562	1134.6509	8.90 × 10^−7^	R.LQLAVDALHR.E
2	714.7017	2141.0832	2141.0753	8.60 × 10^−7^	R.EIGFLEGEINSIEGIHAASR.C
3	482.7427	963.4709	963.4662	0.00072	R.EVDEFIGR.T
4-1	667.8485	1333.6825	1333.6765	7.70 × 10^−6^	R.TPDPFITISSEK.R

Forty micrograms of each protein of the plasma membrane fraction of the wild-type (WT) and *Minute* (*Mi*) (**A**,**B**) and 5 μL of each eluate in the immunoprecipitation experiment of WT and *Mi* (**C**,**D**) were used for LC-MS/MS. Fragments of the trypsin-digested Gγ3 candidates (*p* < 0.05) are shown. The fragment numbers correspond to Figure 4A. Mr(expt) and Mr(calc) correspond to the theoretical molecular mass and the molecular mass that was calculated from the observed molecular mass, respectively. The scores from the Mascot search were 91 (**A**), 90 (**B**), 164 (C), and 248 (**D**).

## Abbreviations

*agb1*	mutant of heterotrimeric G protein β subunit gene in Arabidopsis
*AGB1*	heterotrimeric G protein β subunit gene in Arabidopsis
*AGG1*	heterotrimeric G protein γ1 subunit gene in Arabidopsis
*AGG2*	heterotrimeric G protein γ2 subunit gene in Arabidopsis
*AGG3*	heterotrimeric G protein γ3 subunit gene in Arabidopsis
cMS	crude microsomal fraction
*d1*	mutant of heterotrimeric G protein α subunit gene in rice
*DEP1*	*DENCE AND ERECT PANICLES 1* gene
*DN1*	*DENCE PANICLE 1* gene
*gpa1*	mutant of heterotrimeric G protein α subunit gene in Arabidopsis
*GPA1*	heterotrimeric G protein α subunit gene in Arabidopsis
*GS3*	*GRAIN SIZE 3* gene
*Mi*	*MINUTE*, a mutant of GS3/RGG3
*OsGGC1*	a gene of heterotrimeric G protein γ subunit Type-C in rice, which corresponds to GS3/RGG3
*OsGGC2*	a gene of heterotrimeric G protein γ subunit Type-C in rice, which corresponds to RGG5
*OsGGC3*	a gene of heterotrimeric G protein γ subunit Type-C in rice, which corresponds to which corresponds to DEP1/RGG4
PM	plasma membrane
*RGA1*	heterotrimeric G protein α subunit gene in rice
*RGB1*	heterotrimeric G protein β subunit gene in rice
*RGG1*	heterotrimeric G protein γ1 subunit gene in rice
*RGG2*	heterotrimeric G protein γ2 subunit gene in rice
*RGG3*	heterotrimeric G protein γ3 subunit gene in rice
*RGG4*	heterotrimeric G protein γ4 subunit gene in rice
*RGG5*	heterotrimeric G protein γ5 subunit gene in rice
WB	western blot
WT	wild-type
XLG	extra-large GTP-binding protein
